# Characterizing Plasma-Based Metabolomic Signatures for Metastasis in Non-Small Cell Lung Cancer

**DOI:** 10.3390/metabo15050340

**Published:** 2025-05-20

**Authors:** Manlu Liu, Yanlong Zhu, Sean J. McIlwain, Haotian Deng, Allan R. Brasier, Ying Ge, Michelle E. Kimple, Andrew M. Baschnagel

**Affiliations:** 1 Department of Human Oncology, School of Medicine and Public Health, University of Wisconsin, Madison, WI 53726, USA; mliu243@wisc.edu; 2 Department of Cell and Regenerative Biology, School of Medicine and Public Health, University of Wisconsin, Madison, WI 53726, USA; yzhu353@wisc.edu (Y.Z.); hdeng46@wisc.edu (H.D.); ying.ge@wisc.edu (Y.G.); 3 Human Proteomics Program, School of Medicine and Public Health, University of Wisconsin, Madison, WI 53726, USA; abrasier@wisc.edu; 4 Department of Biostatistics and Medical Informatics, School of Medicine and Public Health, University of Wisconsin, Madison, WI 53726, USA; sean.mcilwain@wisc.edu; 5 Division of Endocrinology, Diabetes, and Metabolism, Department of Medicine, School of Medicine and Public Health, University of Wisconsin, Madison, WI 53726, USA; 6 Department of Chemistry, University of Wisconsin-Madison, Madison, WI 53706, USA; 7 Research Service, William S. Middleton Memorial Veterans Hospital, Madison, WI 53705, USA; 8 University of Wisconsin Carbone Cancer Center, Madison, WI 53792, USA

**Keywords:** lung cancer, metabolomics, biomarkers, clinical study

## Abstract

**Background/Objectives**: The current staging of non-small cell lung cancer (NSCLC) relies on conventional imaging, which lacks the sensitivity to detect micrometastatic disease. The functional assessment of NSCLC progression may provide independent information to enhance the prediction of metastatic risk. The objective of this study was to determine if we could identify a metabolomic signature predictive of metastasis in patients with NSCLC treated with definitive radiation. **Methods**: Plasma samples were collected prospectively from patients enrolled in a clinical trial with non-metastatic NSCLC treated with definitive radiation. Metabolites were extracted, and mass spectrometry-based analysis was performed using a flow injection electrospray (FIE)–Fourier transform ion cyclotron resonance (FTICR) mass spectrometry (MS) method. Early metastasis was defined as metastasis within 1 year of radiation treatment. **Results**: The study cohort included 28 patients. FIE-FITCR produced highly reproducible profiles in technical replicates. A total of 51 metabolic features were identified to be different in patients with early metastasis compared to patients without early metastasis (all adjusted *p*-values < 0.05, Welch’s *t*-test), including glycerophospholipids, sphingolipids, and fatty acyls. In the follow-up samples collected after the initiation of chemotherapy and radiation treatment, a total of 174 metabolic features were significantly altered in patients who developed early metastasis compared to those who did not. **Conclusions**: We identified several distinct changes in the metabolic profiles of patients with NSCLC who developed metastatic disease within 1 year of definitive radiation. These findings highlight the potential of metabolomic profiling as a predictive tool for assessing metastatic risk in NSCLC.

## 1. Introduction

Lung cancer is the leading cause of cancer death in the United States [1]. Non-small cell lung cancer (NSCLC) comprises approximately 85% of all lung cancers [1,2]. The cornerstones of NSCLC treatment involve radiation, surgery, and systemic therapies such as targeted molecular therapy, chemotherapy, and immunotherapy [3]. Even with these therapeutic advances, metastases occur in more than 57% of NSCLC patients [1,2]. Recurrence in distant organs is also the most common type of recurrence, suggesting the disease is already systemic (micrometastatic) at the time of treatment [4,5]. Once metastatic disease is diagnosed, the overall prognosis is poor, with a median survival of 6–18 months [6,7]. Unfortunately, current staging imaging modalities are not sensitive enough to detect micrometastatic disease [8]. Improved staging that assesses micrometastases could help determine prognosis and identify subgroups of patients who would benefit most from tailored therapies.

Metabolomics has emerged as a powerful tool in cancer, alongside proteomics and genomics, with the ability to comprehensively profile cellular metabolic processes that drive tumor progression and metastasis [9,10,11]. Circulating metastatic cells rely on increased glycolysis, disrupted antioxidant metabolism, and altered lipid processing to survive and colonize distant organs [12,13]. These metabolites, detectable in the blood, offer potential as non-invasive biomarkers for predicting metastatic risk. A growing number of metabolomics studies have examined lung-cancer-driven metabolic shifts in a range of biosamples [14]. Metabolomic approaches have successfully differentiated patients with NSCLC from patients without NSCLC [15], distinguished between histological types of lung cancer [16,17], and predicted the prognosis of NSCLC patients on chemotherapy and/or immunotherapy [18,19]. However, metabolite biomarkers have not been used to identify micrometastatic disease in patients with NSCLC.

Fourier transform ion cyclotron resonance (FTICR) mass spectrometry (MS) has emerged as a promising technique for the high-throughput analysis of metabolite samples, especially when coupled with flow injection electrospray (FIE) [20]. We have previously reported a high-throughput FIE-FITCR MS approach that can consistently detect polar and nonpolar metabolites in plasma samples from mice and humans with type 2 diabetes [21,22,23]. This method consistently identified metabolites that predicted type 2 diabetes status and therapeutic response with high reliability and reproducibility [24]. Yet, the application of this technique to plasma samples from human cancer patients, which are more heterogeneous, has not previously been studied.

Here, we examine the metabolite profiles of serial plasma samples from NSCLC patients using comprehensive profiling by FIE-FITCR MS. Metabolite levels were compared between patients who developed early metastasis and those who did not. The results of this pilot study suggest a comprehensive analysis of plasma metabolites may result in a robust diagnostic profile that distinguishes patients at high risk for metastatic NSCLC. This minimally invasive diagnostic would have a high clinical impact by providing therapeutic guidance and improving the standard of care for patients with this potentially devastating disease.

## 2. Materials and Methods

### 2.1. Sample Collection

Blood samples were collected prospectively from 2017 to 2019 at the University of Wisconsin as part of an exploratory endpoint of a clinical trial (NCT02843568) [25]. The study was approved by the University of Wisconsin institutional review board (IRB# 2016-0610 and IRB# 2021-1293), and each patient provided written informed consent before enrollment. Patients had early or localized non-metastatic NSCLC treated with curative intent radiotherapy on a functional lung avoidance trial [25]. Blood was collected prior to treatment (baseline) and after initiation of treatment (follow-up). The follow-up samples were collected at 3 months (17 patients) and at 6 months (5 patients) after radiation treatment. The early metastasis cohort was defined as developing metastases at any site within 1 year of radiation treatment, and the development of early metastases occurred after the collection of the follow-up samples for all patients.

### 2.2. Sample Preparation

Samples were assayed in random order. Metabolite extraction was performed using a 2:1 methanol/plasma extraction, as previously reported [13]. Frozen plasma samples were thawed on ice in a cold room (4 °C). Plasma aliquots (30 μL) were mixed with chilled LC–MS grade methanol (60 μL) followed by vortexing for 10 s. The samples were placed on a nutating mixer for 20 min and then centrifuged at 13,000× *g* for 10 min at 4 °C. The supernatant (60 μL) was transferred to a new microcentrifuge tube and mixed with water (20 μL) for FIE-FTICR MS analysis.

### 2.3. FIE-FTICR Experiment

The FIE-FTICR MS analysis was conducted using a Waters nanoACQUITY ultra-performance liquid chromatography (UPLC) system (Waters Corporation, Milford, MA, USA) linked to a Bruker solariX 12 T FTICR MS (Bruker Daltonics, Bremen, Germany), without an LC column. Metabolite extracts were automatically injected from the UPLC into the FTICR MS through PEEK tubing (100 μm × 40 cm). The injection flow rate was maintained at 15 μL/min. Ion accumulation time was set to 0.1 s, and an 8 M transient size was utilized. Mass spectra were collected over an m/z range of 40–1200, with a Q1 mass of 50 m/z. Each spectrum comprised 50 scans. The dry gas flow was adjusted to 4 L/min at 150 °C. Maximum frequency settings for the octopole (5 MHz), quadrupole (2 MHz), and transfer hexapole (6 MHz) were employed to enhance ion transition. The time of flight was fixed at 0.8 ms, and the sweep excitation power was set to 27%. At 400 m/z, the estimated resolving power was 190,000. The mobile phase consisted of a 50:50 mixture of methanol and water, with 0.1% formic acid added for positive mode and 10 mM ammonium acetate for negative mode. Prior to the experiments, the FTICR MS was calibrated using 1 mM NaTFA in both positive and negative modes.

### 2.4. MS Data Processing

The mass spectra were processed and analyzed using DataAnalysis 4.3 (Bruker Daltonics, Bremen, Germany). The raw mass spectra were processed using ftmsProcessing software V2.3.0 (Bruker Daltonics, Bremen, Germany) to remove Gibbs and harmonic peaks. Mass lists were generated using the T-ReX 2D workflow in MetaboScape 2022 (Bruker Daltonics, Bremen, Germany). The mzDelta was set to 0.50 mDa. The max charge was set to 3, and the intensity threshold was 4 × 10^6^. The bucket lists in the positive and negative modes were merged into one mass list with 1.0 ppm m/z tolerance. Features with a ratio of sample average/blank average < 3 were deleted. Metabolites were included if they were present in at least 5 samples. The chemical formulas were annotated using the SmartFormula function in MetaboScape 2022 with 1.0 ppm as the narrow Δm/z cutoff, 3.0 ppm as the wide Δm/z cutoff, 20 as the narrow mSigma cutoff, and 200 as the wide mSigma cutoff. The metabolites were annotated against HMDB, LipidBlast, MassBank of North America, and Bruker databases based on accurate mass measurement using the same Δm/z cutoff and mSigma cutoff as the SmartFormula annotation.

### 2.5. Statistical Analysis

All analyses were performed using R (v4.4.2). Using MetaboAnalystR (v4.0.0), the data were log-transformed (LogNorm) and scaled using mean centering (MeanCenter). The normalization density and box plots were generated using MetaboanalystR (PlotSampleNormSummary functions). The differential expression values and significance of each metabolite were calculated using Welch’s *t*-test, and the resulting *p*-values were corrected for multiple testing using False Discovery Rate (FDR). Significance was determined by adjusted *p*-value < 0.05 and log2 fold change > 0.6 in terms of the absolute value. Volcano plots were made using the EnhancedVolcano R package. Heatmaps were generated using the NMF R package. Discriminant Partial Least-Squares were performed using the mixOmics R package. Partial Least-Squares Discriminant Analysis (PLS-DA) was performed with two-component models. PLS-DA models were validated with Receiver Operating Characteristic (ROC) Curve Area under the Curve (AUC) and with permutation testing. Mean AUC-ROC (mAUCROC) values from PLS-DA were calculated using 10 repeats of 3-fold cross-validation using code from the mixOmics R package. A total of 999 permutations were calculated using the shuffleSet() call from the permute R package. For each permutation, the mean AUC-ROC was calculated (mAUCROCp) using 10 repeats of 3-fold cross-validation. The permutation *p*-value was calculated using (#(mAUCROCp >= mAUCROC) + 1)/1000, including the original mAUCROC as a permutation.

## 3. Results

### 3.1. Patient Characteristics

The clinical characteristics of the NSCLC patients are summarized in Table 1. This study included 28 NSCLC patients (61% male) with a median age of 69 (range 52–84). Among these patients, 5 developed early metastasis (defined as within 1 year of finishing treatment) and 23 did not develop early metastasis. At the time of diagnosis, 13 patients were classified as stage I, while 15 patients were classified as stage III. The median time to metastasis in the early metastasis patients was 6.2 (range 4.4–9.4) months. Histology included 61% adenocarcinoma, 36% squamous cell carcinoma, and 3% NSCLC not otherwise specified (NOS). All patients had a smoking history, including 39% current smokers and 61% former smokers, at diagnosis. The median survival time was 28.3 months (range 8.5 to 68.3 months). The clinical characteristics of each patient are provided in Supplementary Table S1.

### 3.2. High-Throughput and Reproducible FIE-FTICR MS-Based Platform

An integrated FIE-FITCR MS-based platform [24] was used for the metabolomics-based analysis of the plasma samples. In this high-throughput method, we were able to obtain a high-resolution mass spectrum within 5 min of injection, including a ~2 min wash step. With this setup, 12 samples could be injected during a single hour of instrument time.

To evaluate extraction reproducibility, three aliquots from the same plasma sample were extracted. The mass spectra of the aliquots were highly reproducible (Supplementary Figure S1A,B). In one extraction replicate, a mean of 152 metabolites was able to be annotated by accurate mass (Supplementary Figure S1C). A total of 133 metabolites (87.5%) were shared between the three extraction replicates (Supplementary Figure S1D). The peak intensities of these features shared between the three extraction replicates were highly correlated (coefficients 0.991–0.993) (Supplementary Figure S1E).

**Figure 1:**
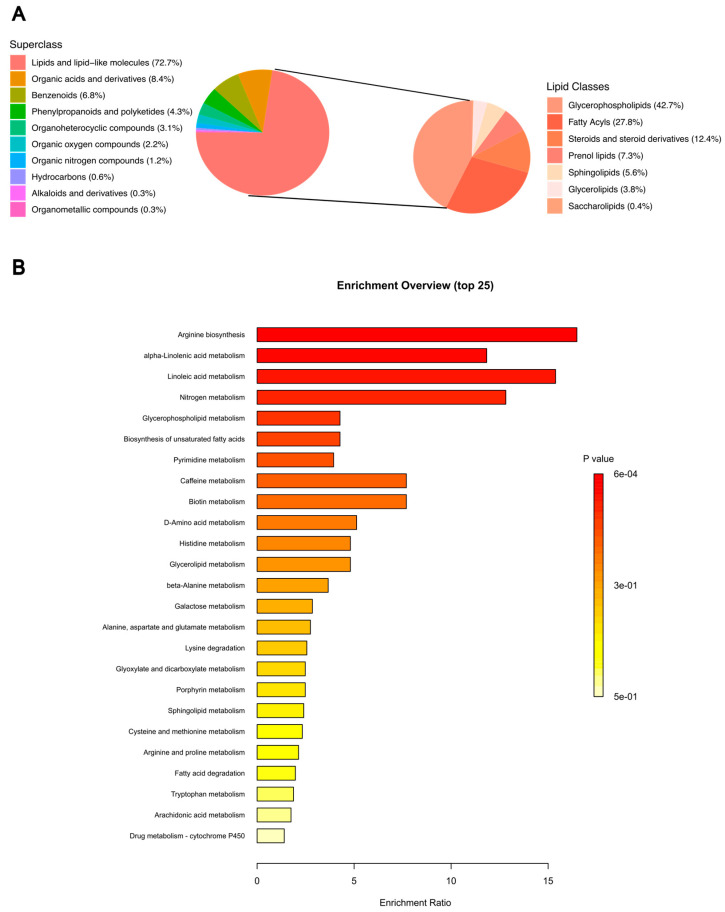
Classes and pathway analysis of identified metabolites in NSCLC plasma samples. (**A**) Superclasses of metabolites identified from the NSCLC plasma samples (left) and classes of lipids identified from NSCLC plasma samples (right). (**B**) Top 25 Kyoto Encyclopedia of Genes and Genomes (KEGG) pathways of metabolites annotated in KEGG database. KEGG—Kyoto Encyclopedia of Genes and Genomes; NSCLC—non-small cell lung cancer.

Across all samples, 1874 metabolic features were detected (Supplementary Tables S2 and S3). A total of 1461 were annotated using SmartFormula. Of these, 379 were annotated by accurate mass, and 82 were annotated in the Kyoto Encyclopedia of Genes and Genomes (KEGG). Both polar and non-polar metabolites were identified from the extraction protocol (Figure 1A). The non-polar metabolites were all included in the lipids and lipid-like molecules superclass, which was the most common superclass represented (72.7% of total metabolites). The polar metabolites were included under multiple superclasses, with the most common ones being organic acids and derivatives (8.4% of total metabolites), benzenoids (6.8% of total metabolites), and phenylpropanoids and polyketides (4.3%). The most common classes within the lipids and lipid-like molecule superclass included glycerophospholipids (42.7%), fatty acyls (27.8%), and steroids and steroid derivatives (12.4%). The most common KEGG pathways were arginine biosynthesis, alpha-linolenic acid metabolism, and linoleic acid metabolism (Figure 1B).

### 3.3. Serial Analysis of Metabolites Reveals Treatment Effects

**Figure 2:**
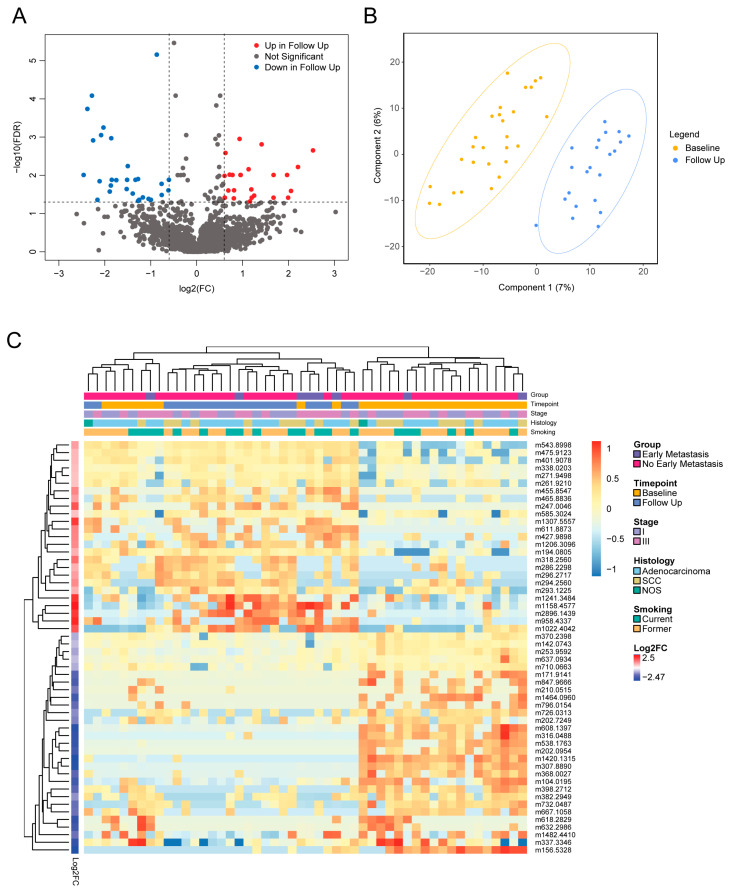
Longitudinal evaluation of metabolites reveals effects of NSCLC treatment. (**A**) Volcano plot of significantly different metabolites in baseline and follow-up samples. (**B**) PLS-DA clustering of baseline and follow-up NSCLC plasma samples. AUROC (mean ± SD): 0.945 ± 0.0168; permutation testing, *p* = 0.001. (**C**) Heatmap with hierarchical clustering of rows and columns represents 54 significant features from Welch’s *t*-test analysis of baseline and follow-up samples (adj *p* < 0.05, log2FC > 0.6). Each row represents a metabolite, and each column represents a patient sample. Rows were annotated with log2FC of normalized metabolite intensity in the baseline group compared to the follow-up group. Columns were annotated with clinical features, including stage, histology, and smoking. log2FC—log2 fold change; NSCLC—non-small cell lung cancer; PLS-DA—Partial Least-Squares Discriminant Analysis; SD—standard deviation.

After the normalization of the 1874 total metabolic features (Supplementary Figure S2), we first characterized the effects of NSCLC treatment. A total of 54 metabolic features were found to be significantly different between baseline and follow-up samples (adj *p* < 0.05, |log2 fold change (log2FC)| > 0.6) (Supplementary Table S4). Of these, 25 metabolites were higher in follow-up samples, and 29 metabolites were lower in follow-up samples (Figure 2A). Seven metabolites were able to be annotated using accurate mass, belonging to the classes of carboxylic acids and derivatives, fatty acyls, prenol lipids, and imidazopyrimidines (Supplementary Table S5). Partial Least-Squares Discriminant Analysis (PLS-DA) revealed that baseline and follow-up samples formed two distinct groups (Figure 2B). The unsupervised hierarchical clustering of the baseline and follow-up samples showed that the baseline and follow-up samples generally formed two separate clusters (Figure 2C). No significant differences in metabolite levels were observed based on stage, smoking, or sex (all adj *p* > 0.05).

### 3.4. Distinct Metabolites Characterize Presence of Early Metastases

**Figure 3:**
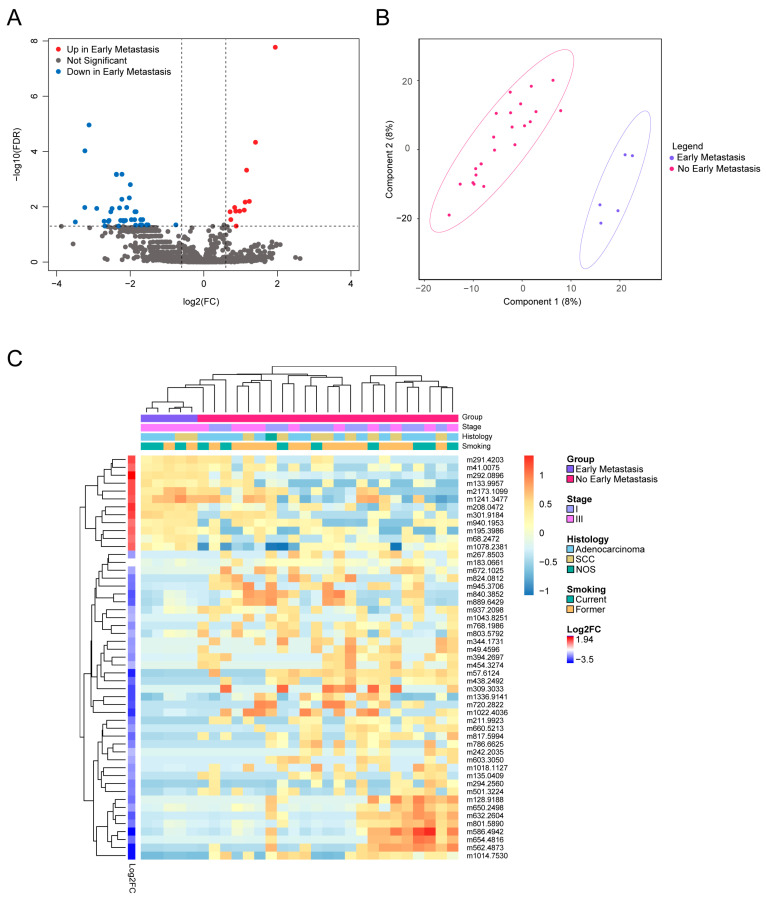
Distinct metabolomics signature in pre-treatment samples separates patients by presence of early metastasis. (**A**) Volcano plot of early metastasis versus no early metastasis baseline samples. (**B**) PLS-DA clustering of early metastasis and no early metastasis baseline samples. AUROC (mean ± SD): 0.687 ± 0.113; permutation testing, *p* = 0.084. (**C**) Heatmap with hierarchical clustering of rows and columns represents 51 significant features from Welch’s *t*-test analysis of early metastasis vs. no early metastasis. Each row represents a metabolite, and each column represents a patient sample. Rows were annotated with log2FC of normalized metabolite intensity in early metastasis group compared to no early metastasis group. Columns were annotated with clinical features, including stage, histology, and smoking. Log2FC—log2 fold change; PLS-DA—Partial Least-Squares Discriminant Analysis; SD—standard deviation.

Since our analysis of baseline and follow-up samples indicated that the metabolome of patients is altered following the initiation of medical and radiation therapy, we evaluated whether metabolites could predict early metastasis, stratified by before and after initiation of treatment. A total of 51 metabolites were found to be significantly different at baseline between patients with early metastasis and those without (adj *p* < 0.05, |log2FC| > 0.6) (Supplementary Table S6). Of these, 39 metabolites were lower in patients with early metastasis, while 12 were elevated compared to patients without early metastasis (Figure 3A). Among these metabolites, nine could be annotated by chemical name (Table 2) and belonged to the following classes: fatty acyls, carboxylic acids and derivatives, diarylheptanoids, organonitrogen compounds, glycerophospholipids, and sphingolipids. All nine metabolites were found to be undetectable in patients with early metastasis compared to those without, reflecting metabolic changes involved in early metastasis. Unsupervised hierarchical clustering further revealed a distinct metabolite signature for patients with early metastasis (Figure 3B). PL-SDA demonstrated that these metabolites form distinct groups based on the presence of early metastasis (Figure 3C).

**Figure 4:**
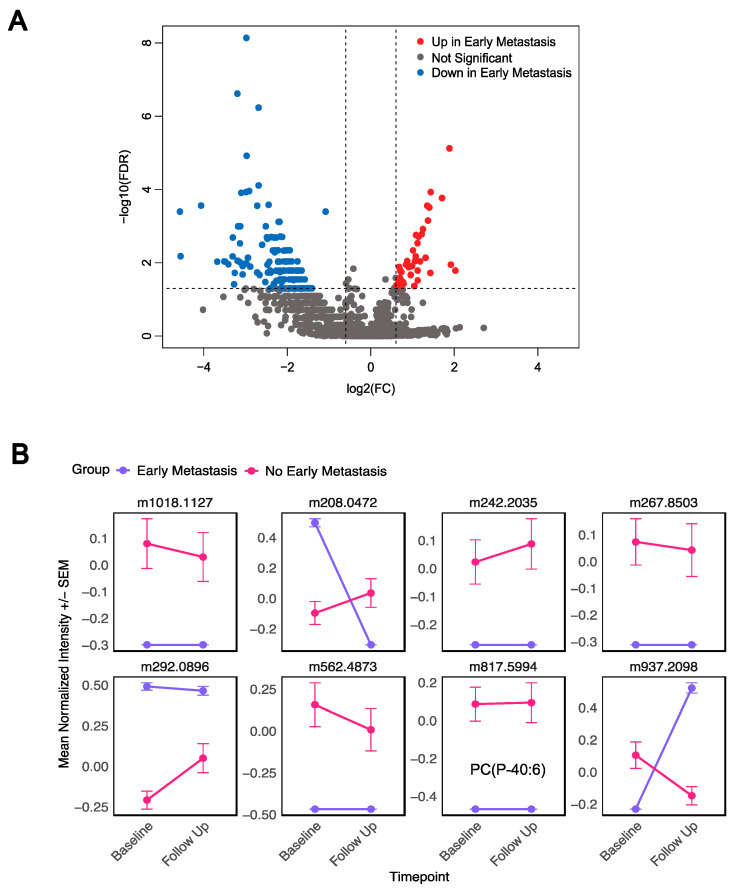
Metabolites in post-treatment samples characterize patients by presence of early metastasis. (**A**) Volcano plot of early metastasis vs. no early metastasis post-treatment samples. (**B**) Eight metabolites that predicted early metastasis in both baseline and follow-up samples.

In the follow-up samples, a total of 174 metabolites were significantly different between early metastasis (*n* = 3) vs. no early metastasis patients (*n* = 18) (adj *p* < 0.05, |Log2FC| > 0.6) (Figure 4A, Supplementary Table S7). A total of 40 metabolites were higher in early metastasis samples, and 134 metabolites were lower in the early metastasis samples compared to samples that did not develop early metastasis. Forty-two metabolites were annotated by accurate mass, with the most abundant classes being fatty acyls (10) and glycerophospholipids (9) (Supplementary Table S8). Eight metabolites that were significantly different at baseline were also different in the follow-up samples (Figure 4B). Out of these eight metabolites, one was able to be annotated as the phosphocholine PC (P-40:6). These findings demonstrate that distinct metabolite signatures characterize the presence of early metastasis prior to and following the initiation of treatment.

## 4. Discussion

This proof-of-principle study aimed to assess the feasibility of using metabolomic profiles to stratify patients likely to progress rapidly from non-metastatic NSCLC to metastatic disease. We identified a signature of 51 metabolite features, differentially abundant in plasma samples from NSCLC patients prior to treatment, in those who developed metastatic disease within one year compared to those who remained metastasis-free during this time frame. In follow-up samples after the initiation of treatment, 174 metabolites were identified as different in patients who developed early metastasis compared to those who did not. Eight features persisted as characterizing early metastasis from baseline to follow-up samples.

The evaluation of metabolite signatures in NSCLC has been limited to predicting prognosis, differentiating between tumor and normal lung tissue, and distinguishing histological subtypes. Multiple studies have identified metabolites that distinguish NSCLC from healthy controls [15,26]. Specifically, a combination of six—metabolites, hypoxanthine, inosine, L-tryptophan, indoleacrylic acid, acylcarnitine C10:1, and lysophosphatidylcholine (18:2)—had a high accuracy of identifying NSCLC from healthy controls [26]. Several other reports found panels of metabolites that predicted prognosis in NSCLC patients undergoing chemotherapy or immunotherapy [19,27]. Additional studies found that levels of phosphocholine, acetate, and glutathione can distinguish between adenocarcinomas and squamous cell carcinomas [28]. However, to our knowledge, this was the first characterization of a metabolite pattern to predict micrometastatic disease in NSCLC.

Lipid metabolism has been implicated in the formation and progression of NSCLC [29]. In particular, tumors modify the lipid membrane for invasion and promote lipid metabolism for energy and for protection from oxidative stress [30]. Accordingly, most of the metabolites annotated in the baseline (66.7%) and follow-up samples (71.4%) were part of lipid pathways. The metabolism of sphingolipids, which are ubiquitously present in cell membranes, is among the most dysregulated processes in NSCLC [31]. Prior reports have found that increased concentrations of sphingolipids in blood serum were positively correlated with overall survival in NSCLC patients [32]. In our study, two sphingomyelins (SM 40:01 and SM d31:1) were undetectable in early metastasis samples prior to treatment, providing evidence of dysregulated sphingolipid metabolism in NSCLC micrometastasis. Besides sphingolipids, glycerophospholipids and derivatives of fatty acids were undetectable in early metastatic samples. In cancer cells, fatty acids are predominantly used to generate glycerophospholipids, the major type of membrane phospholipids [33,34]. Glycerophospholipids in previous studies have been found to distinguish between NSCLC and normal lung tissue [35] and between adenocarcinoma and squamous cell carcinoma [36]. The undetectably low levels of the fatty acid derivatives and glycerophospholipids (glycerophosphoserines, glycerophosphocholines, and glycerophosphoinositols) in samples that developed early metastasis further provide evidence for the reprogramming of cell membrane lipid metabolism in micrometastatic disease. Further research is needed in a larger cohort to validate these findings and explore their potential as clinically relevant biomarkers for micrometastatic NSCLC.

Our findings that metabolites in NSCLC samples differ before and after treatment are consistent with a prior study by Hao et al. [37], which found changes in lung cancer serum metabolites before, during, and after treatment. Despite the effects of treatment on the patient metabolome, we observed distinct metabolite signatures characterizing the development of early metastasis in both treatment-naïve and post-treatment samples. While most of the signature metabolites differed between the pre- and post-treatment timepoints, eight metabolites consistently predicted early metastasis in both baseline and follow-up samples, demonstrating that metabolites can be serially tracked over multiple timepoints to predict early progression to metastatic disease. In addition, the same classes of metabolites (fatty acyls and glycerophospholipids) were altered in both pre- and post-treatment samples, indicating structural lipid membrane changes that continue to be associated with early metastasis throughout chemotherapy and radiation therapy. Future research should focus on the longitudinal monitoring of metabolite levels to identify biomarkers that could facilitate the early detection and monitoring of micrometastatic disease throughout the course of treatment.

Current MS-based metabolomics methods in clinical samples often lack the capability for the fast, reproducible, and simultaneous detection of both polar and nonpolar compounds, with reliable compound annotation. We previously described a high-throughput FIE-FITCR MS metabolomics strategy that can detect changes in both polar and nonpolar metabolites involved in lipid and primary metabolism in a high-throughput manner [21,22,23,24]. While our prior study validated this approach in type 2 diabetes mouse and human plasma samples, we now demonstrate that this strategy can be applied to human NSCLC samples with efficient plasma extraction and reliable metabolite detection. This MS approach overcomes the constraints of many FIE methods that restrict ions to a targeted metabolite list [38]. Furthermore, the ultra-high resolution of the FTICR and selected MS/MS increases the accuracy of the metabolite annotations. In this MS metabolomics approach, we chose to use blood plasma, which has more reproducible metabolite concentrations than serum samples [39]. Our findings demonstrate that blood plasma provides reliable metabolite concentrations that may help classify patients based on their potential for early NSCLC metastasis.

As a pilot study, our work has certain limitations. The small sample size of our cohort restricted the validation of the early metastasis signature in additional patient plasma samples. Moreover, we were not able to control for factors such as diet and co-morbidities that may affect metabolite concentration. However, we determined that the metabolite differences between samples with and without early metastases were independent of stage, sex, smoking, and histology. Further evaluation in a larger cohort is necessary to verify and validate our method for non-invasively staging patients based on the presence of micrometastatic disease.

## 5. Conclusions

With this pilot study, we identified several distinct changes in the metabolic profile of patients with NSCLC who developed early metastatic disease after definitive radiation. Additionally, we demonstrate that our previously reported metabolomics strategy involving high-throughput FIE-FTICR MS, as applied to type 2 diabetes diagnosis and prognosis, can be applied to biosamples from cancer patients, a much more heterogeneous population. This FIE-FTICR MS method has significant potential in the reproducible and efficient metabolomic analysis of clinical samples. These findings underscore the potential of non-invasive metabolomic profiling as a tool for predicting metastatic risk. This study provides insight into the micrometastatic state of NSCLC and reveals a potential metabolite signature that can be validated in larger studies.

## Figures and Tables

**Table 1:** Clinical characteristics of the metabolomics cohort.

Characteristics	N (%)
**Sex**	
Male	17 (61)
Female	11 (39)
**Median age (range) in years**	69 (52–84)
**Stage**	
I	13 (46)
III	15 (54)
**Histology**	
Squamous cell carcinoma	10 (36)
Adenocarcinoma	17 (61)
Not otherwise specified	1 (3)
**Metastasis**	
Early metastasis	5 (18)
No early metastasis	23 (82)
**Smoking Status**	
Current	11 (39)
Former	17 (61)
Non-smoker	0 (0)
**Treatment**	
Radiation only	12 (43)
Radiation + Chemo	6 (21)
Radiation + Chemo/ICI	8 (29)
Radiation + ICI	2 (7)

Chemo: chemotherapy; ICI: immune checkpoint inhibitor.

**Table 2:** Annotated metabolites in pre-treatment samples that characterize patients by the presence of early metastasis.

Mass	Name	Molecular Formula	Class	Subclass	Parent Level 1	Log2FC	Adj *p*
294.256	FA(19:2)	C19H34O2	Fatty acyls	Fatty acids and derivatives	Long-chain fatty acids	−2.174	0.034
344.1731	4-ETHOXYCARBONYL-6-ETHYL-5-METHYL-2-(PARA-TOLYL)PERHYDROPYRROLO(3,4-C)PYRROLE-1,3-DIONE (3A,4-CIS-6,6A-TRANS)	C19H24N2O4	Carboxylic acids and derivatives	Amino acids, peptides, and analogs	Amino acids and derivatives	−1.8881	0.03
720.2822	As-PL(16:0)	C29H58AsO13P	Diarylheptanoids	Linear diarylheptanoids	Linear diarylheptanoids	−2.593	0.034
801.589	PC 36:02 HETE	C44H84NO9P	Glycerophospholipids	Glycerophosphoserines	1-alkyl,2-acyl-glycerol-3-phosphoserines	−2.19	0.032
817.5994	PC(P-40:6)	C48H84NO7P	Glycerophospholipids	Glycerophosphocholines	1-(1Z-alkenyl),2-acyl-glycerophosphocholines	−2.375	0.001
183.0661	Phosphocholine	C5H14NO4P	Organonitrogen compounds	Quaternary ammonium salts	Cholines	−0.759	0.045
632.2604	PI(20:5)	C29H45O13P	Glycerophospholipids	Glycerophosphoinositols	PIs (Phosphatidylinositols)	−2.528	0.015
786.6625	SM(40:1)	C45H91N2O6P	Sphingolipids	Phosphosphingolipids	SMs (Sphingomyelins)	−2.13	0.031
660.5213	SM(d31:1)	C36H73N2O6P	Sphingolipids	Phosphosphingolipids	SMs (Sphingomyelins)	−1.872	0.015

Log2FC—log2 fold change; adj *p*—adjusted *p*-value.

## Data Availability

The raw data supporting the conclusions of this article are available here: https://doi.org/10.5281/zenodo.15128054.

## Supplementary Materials

The following supporting information can be downloaded at: https://www.mdpi.com/article/10.3390/metabo15050340/s1, Supplementary Figure S1: Extraction reproducibility of the NSCLC plasma samples on the FIE-FITCR MS platform; Supplementary Figure S2: Normalization across NSCLC plasma samples; Supplementary Table S1: Clinical characteristics of the patient cohort; Supplementary Table S2: Normalized intensity of the metabolic feature masses across baseline and follow-up samples; Supplementary Table S3: Metabolic features with class annotations; Supplementary Table S4: Welch’s *t*-test of metabolic features across baseline and follow-up samples; Supplementary Table S5: Annotated metabolites significantly different between pre- and post-treatment samples; Supplementary Table S6: Welch’s *t*-test of metabolic features significantly altered between baseline samples from patients with and without early metastasis; Supplementary Table S7: Welch’s *t*-test of metabolic features significantly different in follow-up samples from patients with and without early metastasis; Supplementary Table S8: Annotated metabolites characterizing the presence of early metastasis in post-treatment samples.

## Abbreviations

The following abbreviations are used in this manuscript:

FIE FTICR MS	Flow injection electrospray–Fourier transform ion cyclotron resonance mass spectrometry
KEGG	Kyoto Encyclopedia of Genes and Genomes
Log2FC	Log2 fold change
PLS-DA	Partial Least-Squares Discriminant Analysis
