# Supplementary material for: Characterizing Plasma-Based Metabolomic Signatures for Metastasis in Non-Small Cell Lung Cancer
Source: Metabolites. 2025 May 20;15(5):340. doi: 10.3390/metabo15050340 (PMC12113581; doi:10.3390/metabo15050340)
Supplement: Supplementary file 1 [file metabolites-15-00340-s001.zip › Supplementary Figures_5_19_25.pdf]

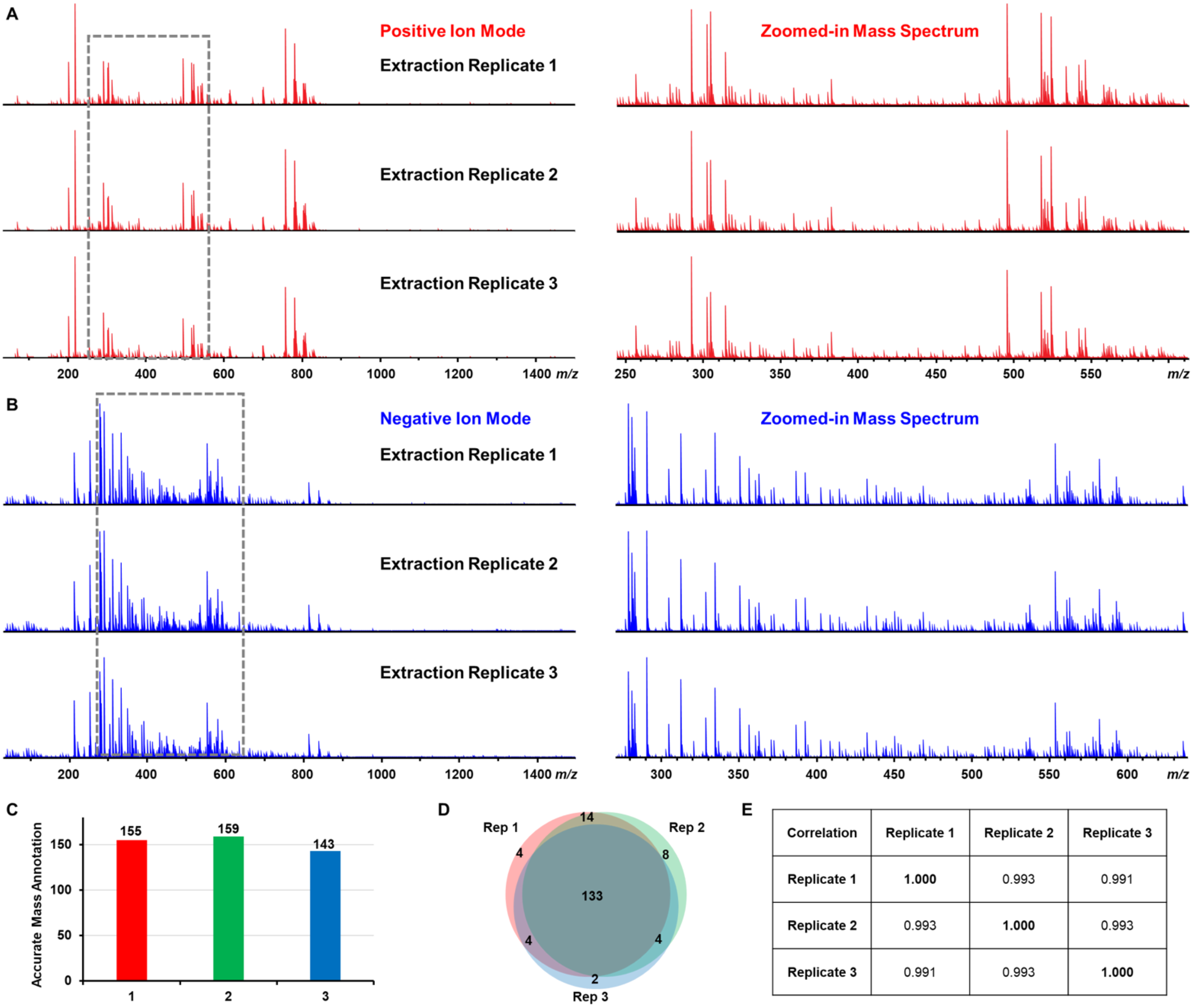

Correlation	Replicate 1	Replicate 2	Replicate 3
Replicate 1	**1.000**	0.993	0.991
Replicate 2	0.993	**1.000**	0.993
Replicate 3	0.991	0.993	**1.000**

Supplementary Figure S1. Extraction reproducibility of the NSCLC plasma samples on the FIE-FITCR MS platform.

(A) Full and zoomed-in mass spectra of three extraction replicates in positive ion mode. (B) Full and zoomed-in mass spectra of three extraction replicates in negative ion mode. (C) Accurate mass annotation numbers of three extraction replicates. (D) Venn diagram of accurate mass annotations of three extraction replicates. (E) Correlation analysis of three extraction replicates.

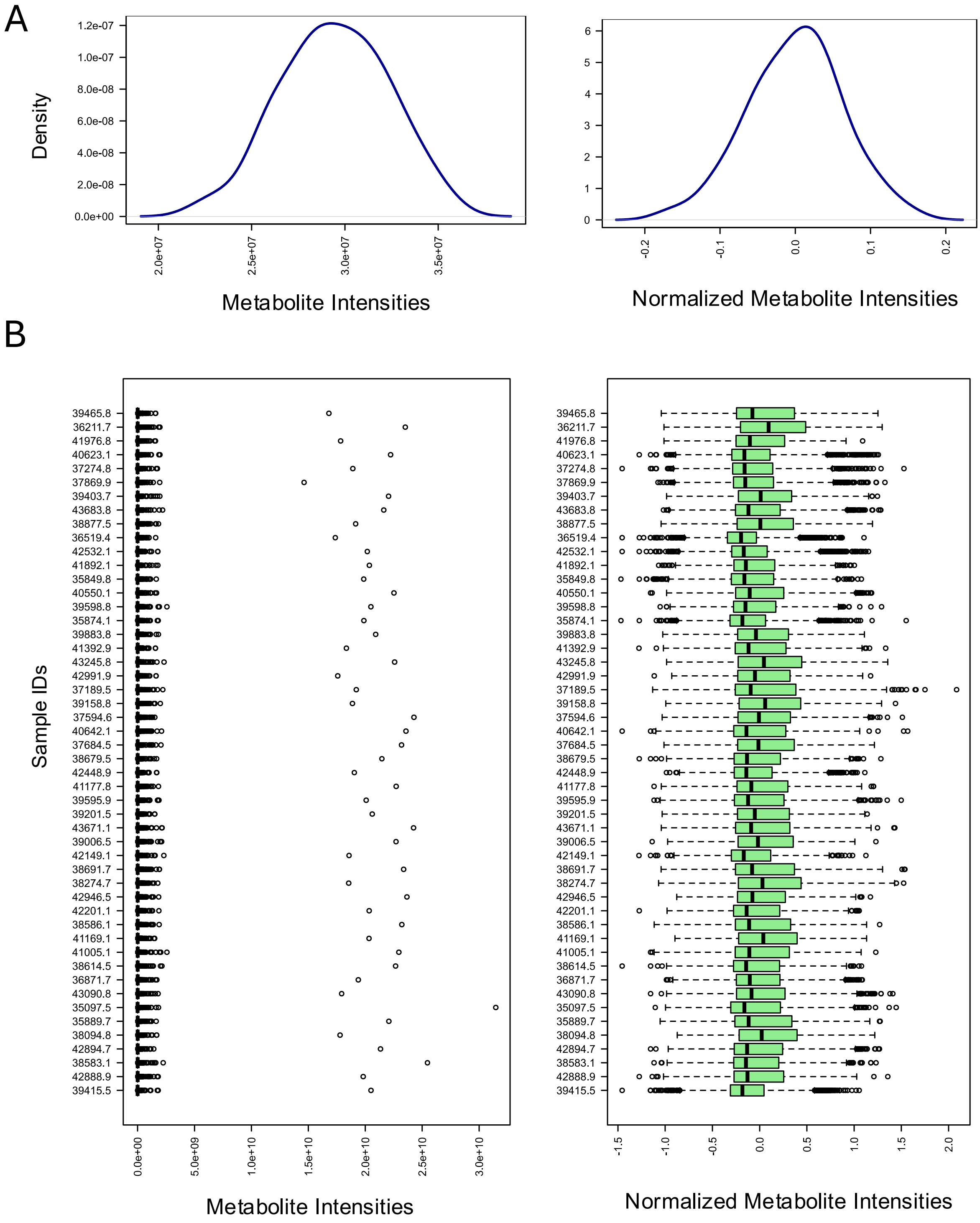

Supplementary Figure S2. Normalization across NSCLC plasma samples.

(A) Density plots depicting distribution of the metabolite intensities before and after applying log transformation and mean centering. (B) Box plots of the sample intensities before and after applying log transformation and mean centering.
